# Inhibition of Histone H3K27 Acetylation Orchestrates Interleukin-9-Mediated and Plays an Anti-Inflammatory Role in Cisplatin-Induced Acute Kidney Injury

**DOI:** 10.3389/fimmu.2020.00231

**Published:** 2020-03-03

**Authors:** Wenjuan Jiang, Xinrong Yuan, Hong Zhu, Changsheng He, Caiqiong Ge, Qing Tang, Chuanting Xu, Bingfeng Hu, Cheng Huang, Taotao Ma

**Affiliations:** ^1^Anhui Province Key Laboratory of Major Autoimmune Diseases, School of Pharmacy, Anhui Institute of Innovative Drugs, Anhui Medical University, Hefei, China; ^2^Xiangya School of Medicine, Central South University, Changsha, China; ^3^College of Pharmacy, Northeast Ohio Medical University, Rootstown, OH, United States

**Keywords:** interleukin-9, H3K27Ac, inflammation, macrophage, acute kidney injury, cisplatin

## Abstract

Nephrotoxicity is a major side effect of cisplatin (CP)- and platinum-related chemotherapy, and inflammation contributes to disease pathogenesis. Interleukin-9 (IL-9) is a pleiotropic cytokine associated with inflammation. Here, we investigated the key role of IL-9 as a regulator of protective mechanisms in CP-induced acute kidney injury (AKI). We observed that IL-9 was decreased not only in a CP-induced AKI mouse model but also in THP-1 and RAW264.7 cell lines. Seventy-two hours post-CP injection, renal dysfunction and tubule injury were significantly attenuated in IL-9 overexpression adeno-associated virus 9 (AAV9)-treated mice. The levels of serum urea, serum creatinine, kidney injury molecule-1 (KIM-1), and histological damage were partially diminished following treatment with IL-9. The renoprotective effects of IL-9 may be attributed to the regulation of cytokines, and we found that IL-9 acted on macrophages in a regulatory manner, promoting an anti-inflammatory phenotype. Furthermore, IL-9 enhanced the suppression of macrophage-driven renal inflammation. Inhibition of H3K27 acetylation orchestrated IL-9-mediated renoprotection in CP-induced AKI. Thus, our findings indicate novel and potent anti-inflammatory properties of IL-9 that confer preservation of kidney function and structure in CP-induced AKI, which may counteract kidney disease procession.

## Introduction

Cisplatin (CP) is a potent antineoplastic agent that is widely used to treat various types of tumors (1, 2). However, its clinical application is limited by time- and dose-dependent nephrotoxicity, which is associated with high morbidity and mortality (3, 4). Inflammation is reported to be involved in the development and amplification of kidney injury (5, 6). Recent studies revealed that activation of tumor necrosis factor-α (TNF-α) and interleukin-6 (IL-6) plays a decisive role in nephrotoxicity by inducing a variety of signaling pathways. These factors trigger the release of pro-inflammatory cytokines and cell infiltration, which aggravate kidney injury (7–9).

In recent years, a number of studies have broadened our understanding of IL-9 involvement in tissue inflammation. Kearley et al. (10) reported that IL-9 aggravates inflammation in helminth-induced lung inflammation, whereas blocking IL-9 ameliorates lung inflammation. Nalleweg et al. (11) suggest that the targeting of IL-9 may become a therapeutic option for patients with ulcerative colitis (UC). However, Rauber et al. (12) identified IL-9 as a master regulator for the resolution of arthritis by inducing inflammation. As a pleiotropic cytokine, IL-9 is involved in several diseases like cancer and autoimmune diseases (13). Due to conflicting reports in the literature, the contribution of IL-9 to disease is unclear, especially in the context of CP-induced acute kidney injury (AKI).

Recently, we identified that histone deacetylase 2 (HDAC2) is necessary to maintain apoptosis in AKI (14, 15). Chromatin regulation through histone modification plays an essential role in coordinating the expression of multiple genes. Alterations in chromatin induced by histone modifiers and readers regulate critical transcriptional programs involved in both normal development and tumor differentiation (16, 17). Lloyd and Harker (18) reported that acetylation of histones mediates IL-9 expression in asthma. Inhibition of H3K27 acetylation could decrease the expression of IL-9. Xiao et al. (19) also found that acetylation of histones (specifically, H3K27) promoted transcription of IL-9. H3K27 is a specific histone protein (H3) that is acetylated at lysine 27 (H3K27Ac), a marker of transcriptional activity. Thus, the inhibition of H3K27 acetylation may orchestrate the expression of IL-9 in kidney diseases.

The aim of this study was to determine whether IL-9 can suppress CP nephrotoxicity, and if so, we aimed to determine the underlying mechanisms. Our results demonstrate that IL-9 acts on macrophages toward a regulatory manner, inducing an anti-inflammatory phenotype and enhancing the suppressive function of macrophage-driven renal inflammation. Inhibition of H3K27 acetylation orchestrates IL-9-mediated renoprotection in CP-induced AKI. Our findings highlight IL-9 as a potential therapeutic for targeting inflammation in AKI.

## Materials and Methods

### Experimental Reagents

Trichostatin A (TSA), dimethyl sulfoxide (DMSO), valproate (VPA), and CP were obtained from Sigma Aldrich (St. Louis, MO, USA). CP is 1 mg/ml solution in sterile normal saline. C646 was purchased from Apexbio (USA). β-actin and kidney injury molecule-1 (KIM-1) primary antibodies and goat anti-rabbit or anti-mouse immunoglobulin G (IgG) horseradish peroxidase (HRP) secondary antibodies were purchased from Bioss (Beijing, China). H3K27Ac antibody was obtained from Abcam (UK). Human recombinant IL-9 was purchased from Bioworld Technology (Nanjing, China). Creatinine (Cr) and blood urea nitrogen (BUN) assay kits were purchased from Njjcbio (Nanjing, China). ELISA kits for detection of IL-9 and TNF-α were purchased from Jymbio (Wuhan, China).

### Animals and Drug Administration

C57BL/6 mice, supplied by the Experimental Animal Centre of Anhui Medical University, were used to establish the AKI model. Animal work took place in Anhui Province Key Laboratory of Major Autoimmune Diseases at Anhui Medical University. These mice were maintained under specific pathogen-free conditions at 22°C under a 12-h light–dark cycle and received standard chow and water *ad libitum*. All animal experiments were performed in accordance with the Regulations of the Experimental Animal Administration issued by the State Committee of Science and Technology of China. All animal procedures were approved by the Animal Experimentation Ethics Committee from Anhui Medical University, Anhui, China (LLSC20190277). Animals and all experiments used protocols approved by the institutions' subcommittees on animal care. All efforts were made to reduce animal suffering and to minimize the number of animals used.

### Cisplatin Administration to Mice

Mice were administered CP (MilliporeSigma; 1 mg/ml solution in sterile normal saline) or normal saline in a single intraperitoneal (i.p.) injection. Mice were sacrificed 72 h after administration of CP, and tissue and blood were collected for further analysis.

### Adeno-Associated Virus 9 Mouse Model

Mice were divided into five groups with six animals in each group. Vehicle group mice were administered normal saline in a single i.p. injection. CP group mice were administered CP at 20 mg/kg in a single i.p. injection. Adeno-associated virus 9 (AAV9) group mice were injected with an empty AAV9 vector by tail vein injections and administered normal saline in a single i.p. injection. AAV9-IL9 group mice were injected with an AAV9-IL9 vector by tail vein injections and administered normal saline in a single i.p. injection. AAV9-IL9+CP group mice were injected with an AAV9-IL9 vector by tail vein injections and administered CP at 20 mg/kg in a single i.p. injection. The AAV9 tail vein injection method was followed as previously described (20). Animals were sacrificed 72 h after injection of CP, and blood and kidney tissues were collected. Kidney tissues were processed for histology, F4/80 assay, and protein or RNA isolation.

### Histone Deacetylase Inhibitors Mouse Model

The mice were randomly divided into five groups (*n* = 6 per group): Vehicle group mice were administered normal saline in a single i.p. injection. CP group mice were administered CP at 20 mg/kg in a single i.p. injection. TSA+CP group mice were treated with TSA (1 mg/kg body weight) *via* gavage. VPA+CP group mice were treated with VPA (1 mg/kg body weight) *via* gavage. C646+CP group mice were injected i.p. with 10 μg of C646 in 0.5 ml of PBS. The last three groups were treated every 24 h for 2 days before CP at 20 mg/kg in a single i.p. injection.

### Creatinine and Blood Urea Nitrogen Assay Kits

We determined the concentrations of Cr and BUN in serum from C57BL/6 AKI mice *via* Cr and BUN assay kits according to the manufacturer's instructions. The creatinine clearance (CCr) was calculated using this equation: CCr (ml/min kg) = UCr × 24 h UV/(SCr × body weight × 24 × 60).

### Histopathology

Renal tissues of mice were fixed in 4% paraformaldehyde for 24 h immediately following killing, processed for histological examination according to a conventional method, and stained with H&E and F4/80. Ten fields of ×10 and × 40 original magnifications were examined and averaged. The slides were scored in a blinded manner and de-identified.

### Cell Culture

Human renal proximal tubule cells (HK-2 cells) were kindly provided by Prof. Huiyao Lan (Li Ka Shing Institute of Health Science, Hong Kong, China). These cells were cultured in DME/F-12 (HyClone, Logan, UT, USA) supplemented with 10% (vol/vol) heat-inactivated fetal bovine serum (FBS; Merck Millipore, Darmstadt, Germany) at 37°C in a humidified incubator under 5% carbon dioxide (CO_2_). THP-1 cells, a human leukemia monocytic cell line, which have been extensively used to study monocyte/macrophage functions, were cultured in RPMI 1640 (HyClone, Logan, UT, USA) supplemented with 10% (vol/vol) heat-inactivated FBS (Merck Millipore, Darmstadt, Germany) at 37°C in a humidified incubator under 5% CO_2_. RAW264.7 cells were obtained from the Type Culture Collection of the Chinese Academy of Sciences (Shanghai, China) and were maintained in Dulbecco's modified Eagle's medium (DMEM, Gibco, USA) supplemented with 10% FBS (Gibco, USA) and incubated at 37°C in an atmosphere of 5% CO_2_.

### THP-1 Conditioned Medium

THP-1 cells (~1.2 × 10^6^ cells/cm^2^) were divided into three groups: Normal group, CP group, and CP+IL-9 group. THP-1 conditioned medium (CM) was set up as described: THP-1 cells (~1.2 × 10^6^ cells/cm^2^) were incubated with phorbol 12-myristate 13-acetate (PMA) (100 nmol/L) for 24 h to allow differentiation to macrophages, before washing with PBS. Cells were then incubated with RPMI 1640 medium without FBS, treated with 100 ng/ml of IL-9 (CP+IL-9 group) or without 100 ng/ml of IL-9 (CP group) for 8 h, before adding CP (100 μmol/L) for a further 8 h.

We harvested CM of macrophages and added to HK-2 cells for 24 h while vehicle group cells were cultured with regular medium. Cells were harvested and subjected to Western blot and immunofluorescence staining.

### ELISA

THP-1 or RAW264.7 cells were treated with CP (100 μmol/L) for 8 h to evaluate the concentration of IL-9 in the supernatant, while normal group cells were treated with normal saline *via* ELISA commercialized protocol. Furthermore, the THP-1 or RAW264.7 cells above were administrated with 100 ng/ml of recombinant IL-9 (rIL-9) for 8 h, harvested supernatant for TNF-α ELISA. The concentration of IL-9 in the kidneys of AKI mice was also measured *via* ELISA using a commercialized protocol.

### Histone Deacetylase Inhibitors With THP-1 and RAW264.7 Cells

To determine CP-mediated downregulation of H3K27Ac expression and induce the decrease of IL-9, cells were treated with CP or CP plus specific inhibitor (0.1 μmol/L of TSA or 1 mmol/L of VPA) for 24 h. Cells were harvested for protein or RNA isolation. Cells and supernatants were harvested and subjected to cytokine analysis.

### Immunofluorescence Staining

Sections were blocked with 10% bovine serum albumin (BSA) solution to avoid non-specific staining. Sections were incubated with rabbit polyclonal primary antibodies against KIM-1 (1:500). Sections were incubated overnight at 4°C, followed by incubation with anti-rabbit Cy-3 (1:200)-conjugated secondary antibodies and nuclei staining with 4′,6-diamidino-2-phenylindole (DAPI; Beyotime Biotechnology, Shanghai, China). Stained sections were examined with an inverted fluorescence microscope (Carl Zeiss Axio Vert.A1, Jena, Germany).

### Acridine Orange–Ethidium Bromide Staining

Apoptotic morphology of treated cells was assessed and distinguished by acridine orange–ethidium bromide (AO/EB) fluorescent staining. The staining method was performed *via* introduction of a mixture of fluorescent dyes, AO/EB in a 1:1 ratio on treated cells. THP-1 cells and RAW264.7 cells (2 × 10^3^ cells/well) were seeded in chamber slides. The adherent cells were washed with 200 μl of PBS, and 2 μl of the dye mixture containing ethidium bromide (100 mg/ml) and acridine orange (100 mg/ml) in 1:1 ratio was placed on each well of the chamber slide. Chamber slides were examined immediately under a fluorescence microscope (Carl Zeiss Axio Vert.A1, Jena, Germany).

### Western Blot

Whole protein extracts were separated by 10 or 12% sodium dodecyl sulfate polyacrylamide gel electrophoresis (SDS-PAGE) and transferred to polyvinylidene difluoride (PVDF) membranes, which were incubated with primary antibodies against KIM-1 (1:500; Santa Cruz Biotechnology, Dallas, TX, USA). The membranes were then washed in TBS/Tween 20 and incubated with secondary antibodies correspondingly. After extensive washing in TBS/Tween 20, protein bands were visualized with an ECL chemiluminescent kit (ECL-plus; Thermo Fisher Scientific, Pittsburgh, PA, USA).

### Real-Time Reverse Transcriptase-PCR

Total RNA was collected from kidney tissues, THP-1 and RAW264.7 cells, using TRIzol reagents (Invitrogen). First-strand cDNA was synthesized using the Thermoscript RT-PCR synthesis kit (Fermentas, Pittsburgh, PA, USA) according to the manufacturer's instructions. Real-time quantitative PCR analyses for mRNA were performed by using Thermoscript RT-qPCR kits (Fermentas, Pittsburgh, PA, USA) in an ABI PRISM StepOnePlus Real-Time PCR System (Applied Biosystems, Foster City, CA, USA). The products were used as templates for amplification using the SYBR Green PCR amplification reagent (Qiagen, Valencia, CA, USA) and gene-specific primers. Relative expression levels were calculated according to the standard 2^−ΔΔ*C*^t method (21). The forward and reverse primers used for PCR were as follows:

IL-9 (human) forward: 5′-ATGGTCCTTACCTCTGCCCT-3′; reverse: 5′-GGCACTTGGAAGCTGGATCT-3′; IL-9 (mouse) forward: 5′-TCTTCAGTTCTGTGCTGGGC-3′; reverse: 5′-CAGCTGCATTTTGACGGTGG-3′;glyceraldehyde-3-phosphate dehydrogenase (GAPDH) forward: 5′-CCCAGCAAGGACACTGAGCAAG-3′; and GAPDH reverse: 5′-GGTCTGGGATGGAAA TTGTGAGGG-3′. The expression of GAPDH was used as an internal control (22).

### Statistical Analysis

Data were expressed as the mean ± SEM. Statistical significance was analyzed using one-way analysis of variance (ANOVA), followed by Tukey *post hoc* tests using GraphPad Prism 5 software (GraphPad, La Jolla, CA, USA), and differences were considered significant where *P* < 0.05.

## Results

### Interleukin-9 Is Downregulated in Cisplatin-Induced Acute Kidney Injury

**Figure 1:**
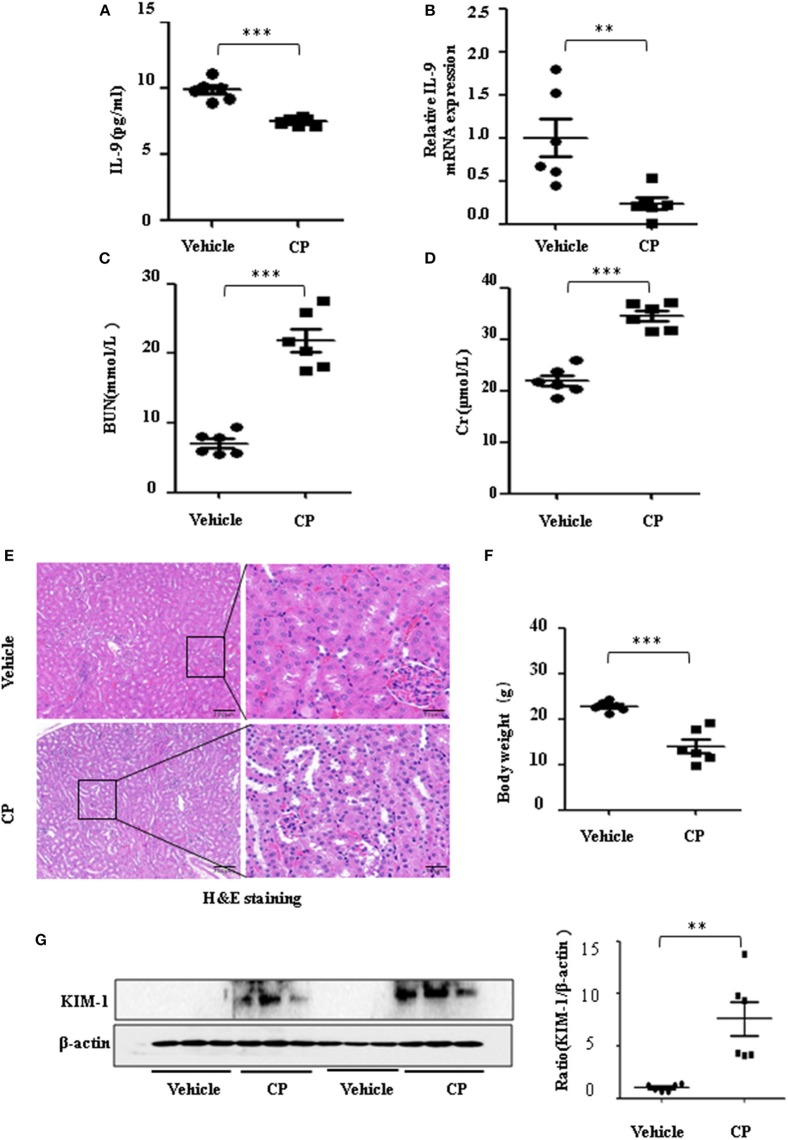
The secretion of interleukin-9 (IL-9) was downregulated in the cisplatin (CP)-induced acute kidney injury (AKI) model. Mice were induced with a single intraperitoneal injection of either saline (vehicle) or CP (20 mg/kg body weight). Three independent experimental series were performed with six animals per group in each series. Data were represented as mean ± SD (*n* = 6). **(A)** The level of IL-9 in the kidneys was assessed by ELISA. ***P < 0.001 vs. the vehicle group. **(B)** The mRNA level of IL-9 in the kidneys of CP-induced AKI. Dot plots showed the corresponding quantification of mRNA levels and fold change. **P < 0.01 vs. the vehicle group. **(C)** Changes in blood urea nitrogen (BUN) level with CP-induced mice. ***P < 0.001 vs. the vehicle group. **(D)** Changes in serum creatinine (Cr) level with CP-induced mice. ***P < 0.001 vs. the vehicle group. **(E)** Representative images of H&E staining of the kidney section of CP-induced mice were analyzed under bright–field microscopy. Magnification: ×10 and ×40. **(F)** Body weight in each group after 72 h of CP injection. ***P < 0.001 vs. the vehicle group. **(G)** Western blot was performed on the CP-induced kidney to determine the kidney injury molecule-1 (KIM-1) expressions. Each lane represents one sample from an individual mouse. Dot plots show the corresponding quantification of band intensity and fold change.

To determine the contribution of IL-9 to CP-induced AKI, we first assessed the expression level of IL-9 in kidneys following CP exposure. As shown in Figure 1A, relative to the vehicle group, the serum level of IL-9 was downregulated 1.3-fold in CP-induced mice. IL-9 mRNA levels were downregulated in the kidneys more than 4-fold at 72 h in CP-induced mice (Figure 1B) in contrast with significantly increased levels of BUN and serum Cr (Figures 1C,D). Histologic evidence of injury was assessed in kidneys from mice subjected to CP and showed marked renal tubular injury as reflected by the edema of renal tubular epithelial cells, dilation of renal capsule cavity, and the epithelial cells of the local focal necrosis collapse (Figure 1E). Body weight, an indicator of health, also declined after 72 h of CP injection (Figure 1F). Likewise, the protein level of KIM-1, an established biomarker for renal proximal tubule injury, was barely detectable in the kidney of vehicle mice but increased dramatically after CP injection (Figure 1G).

### Interleukin-9 Mediates Cisplatin-Induced Acute Kidney Injury

**Figure 2:**
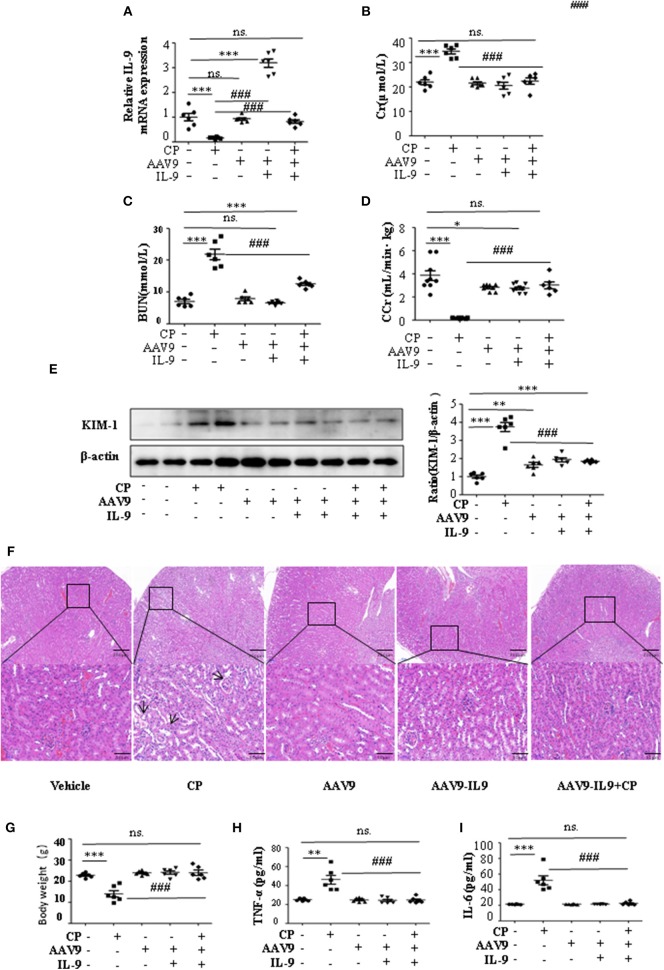
Interleukin-9 (IL-9) alleviated cisplatin (CP)-induced acute kidney injury. Mice were injected with adeno-associated virus 9 (AAV9)-IL9 vectors by tail vein injections to confirm the overexpression of IL-9 in kidneys then induced by CP (20 mg/kg) as the AAV9-IL9+CP group. Mice were injected with AAV9-IL9 vectors by tail vein injections without CP injection as the AAV9-IL9 group. Mice were injected with AAV9 empty vectors by tail vein injections without CP injection as the AAV9 group. Mice were induced with a single intraperitoneal injection of CP as the CP group. Mice were given a single intraperitoneal injection of saline as the vehicle group. Three independent experimental series were performed with six animals per group in each series. Data were represented as mean ± SD (*n* = 6). **(A)** The mRNA level of IL-9 in the kidneys in each treatment group. Dot plots show the corresponding quantification of mRNA level and fold change. ***P < 0.001 vs. the vehicle group. ^###^*P* < 0.001 vs. the CP group. **(B)** Changes in the serum creatinine (Cr) level with each treatment group. ***P < 0.001 vs. the vehicle group; ^###^*P* < 0.001 vs. the CP group. **(C)** Changes in the serum blood urea nitrogen (BUN) levels with each treatment group. ***P < 0.001 vs. the vehicle group; ^###^*P* < 0.001 vs. the CP group. **(D)** Changes in the creatinine clearance rate (CCr) level with each treatment group. ***P < 0.001, *P < 0.05 vs. the vehicle group; ^###^*P* < 0.001 vs. the CP group. **(E)** Western blot was performed on different treatment groups to determine the kidney injury molecule-1 (KIM-1) expressions. Each lane represents one sample from an individual mouse. Dot plots showed the corresponding quantification of band intensity and fold change. ***P < 0.001, **P < 0.01 vs. the vehicle group; ^###^*P* < 0.001 vs. the CP group. **(F)** Representative images of H&E staining of the kidney section of the different groups of mice after 72 h of CP injection. Magnification: ×10 and ×40. **(G)** Body weight of mice in each group after 72 h of CP injection. ***P < 0.001 vs. the vehicle group. ^###^*P* < 0.001 vs. the CP group. **(H)** The level of pro-inflammatory cytokine tumor necrosis factor-α (TNF-α) in the serum of mice from each group after 72 h of CP injection. **P < 0.01 vs. the vehicle group; ^###^*P* < 0.001 vs. the CP group. **(I)** The level of pro-inflammatory cytokine interleukin-6 (IL-6) in the serum of mice from each group after 72 h of CP injection. ***P < 0.001 vs. the vehicle group; ^###^*P* < 0.001 vs. the CP group.

To determine whether IL-9 was involved in AKI, we assessed kidney function 72 h after CP administration in wild-type (WT) and IL-9 overexpression mice. As recombinant AAV (rAAV) system has been proved to be safe for therapeutic payload delivery (23), we chose AAV9 vectors delivering IL-9 to CP-induced AKI mice by tail vein injections. A significant increase of IL-9 mRNA level was seen in the kidneys of the mice treated with AAV9-IL9 (Figure 2A). As expected, injection of mice with CP (20 mg/kg) led to severe kidney dysfunction, as reflected by elevated serum Cr and BUN concentrations. In contrast, Cr and BUN levels were significantly decreased in AAV9-IL9 mice after CP injection (Figures 2B,C). Consistent with the levels of Cr and BUN, the Cr clearance (CCr) was also calculated (Figure 2D). Western blot analysis of kidney tissues indicated that the protein level of KIM-1 was also downregulated 1.4-fold in AAV9-IL-9 mice after CP injection (Figure 2E).

The improvement in renal function was also reflected in less severe histologic damage (Figure 2F). CP resulted in severe tubular injury reflected by sloughing of tubular epithelial cells and dilation of tubules. These changes were absent in kidneys from mice injected with CP and AAV9-IL9.

Similarly, body weight also changed (Figure 2G). Excessive secretion of pro-inflammatory cytokines is associated with exacerbated kidney injury. To identify the role of IL-9 in pro- and anti-inflammatory cytokine secretion, we performed ELISAs. The results are summarized in Figures 2H,I and indicate that CP induces increased TNF-α and IL-6 levels, but these increases were significantly blunted in the serum of AAV9-IL9 mice.

### Interleukin-9 Suppresses Pro-inflammatory Cytokines Associated With Macrophages

**Figure 3:**
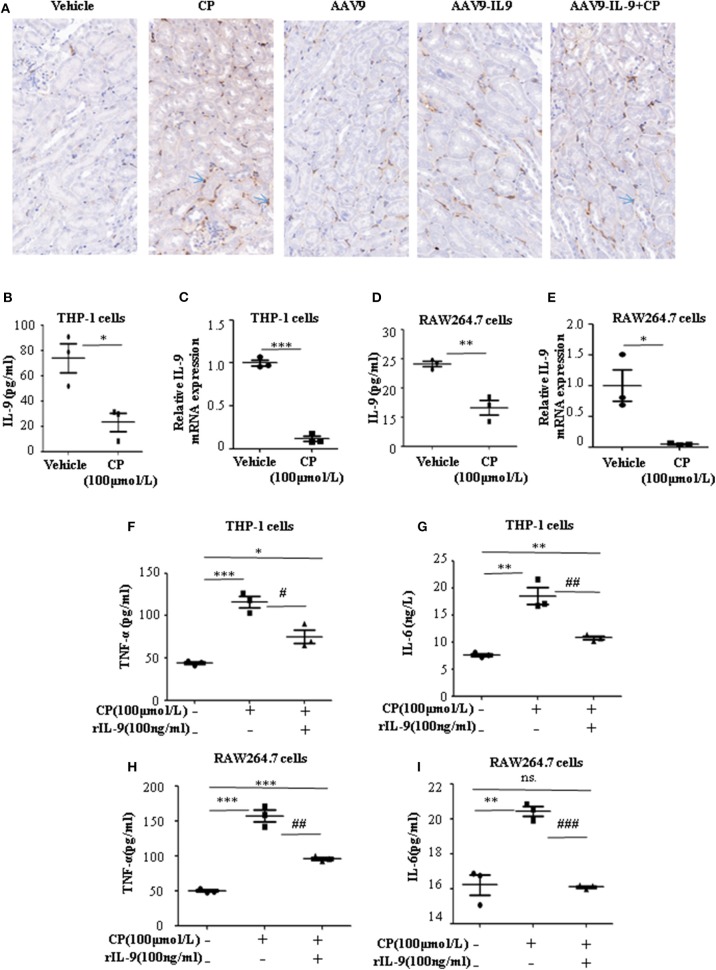
Interleukin-9 (IL-9) suppressed the pro-inflammatory cytokines associated with macrophages *in vivo* and *in vitro*. Data were represented as mean ± SD of three independent experiments. **(A)** Representative images of immunohistochemistry staining infiltrated macrophages of different groups of mice. Mice were injected with adeno-associated virus 9 (AAV9)-IL9 vectors by tail vein injections to confirm the overexpression of IL-9 in the kidneys then induced by cisplatin (CP) (20 mg/kg) as the AAV9-IL9+CP group. Mice were injected with AAV9-IL9 vectors by tail vein injections without CP injection as the AAV9-IL9 group. Mice were injected with AAV9 empty vectors by tail vein injections without CP injection as the AAV9 group. Mice were induced with a single intraperitoneal injection of CP as the CP group. Mice were given a single intraperitoneal injection of saline as the vehicle group. Magnification: ×40. **(B)** The level of IL-9 in the supernatant of THP-1 cells stimulated with CP (100 μmol/L) for 8 h. *P < 0.05 vs. the vehicle group. **(C)** The mRNA level of IL-9 in THP-1 cells stimulated with CP (100 μmol/L) for 8 h. Dot plots showed the corresponding quantification of mRNA level and fold change. ***P < 0.001 vs. the vehicle group. **(D)** The levels of IL-9 in the supernatant of RAW264.7 cells with CP (100 μmol/L) administration. **P < 0.01 vs. the vehicle group. **(E)** The mRNA levels of IL-9 in RAW264.7 cells with CP (100 μmol/L) administration. Dot plots showed the corresponding quantification of mRNA level and fold change. *P < 0.05 vs. the vehicle group. **(F)** The level of tumor necrosis factor-α (TNF-α) in the supernatant of THP-1 cells stimulated with CP (100 μmol/L) and treated with 100 ng/ml recombinant IL-9. *P < 0.05, ***P < 0.001 vs. the vehicle group; ^#^*P* < 0.05 vs. the CP-treated group. **(G)** The level of IL-6 in the supernatant of THP-1 cells stimulated with CP (100 μmol/L) and treated with 100 ng/ml recombinant IL-9. **P < 0.01 vs. the vehicle group; ^##^*P* < 0.01 vs. the CP-treated group. **(H)** The level of TNF-α in the supernatant of RAW264.7 cells stimulated with CP (100 μmol/L) and treated with 100 ng/ml recombinant IL-9. ***P < 0.001 vs. the vehicle group; ^##^*P* < 0.01 vs. the CP-treated group. **(I)** The level of IL-6 in the supernatant of RAW264.7 cells stimulated with CP (100 μmol/L) and treated with 100 ng/ml recombinant IL-9. **P < 0.01 vs. the vehicle group; ^###^ *P* < 0.001vs. the CP-treated group.

Infiltration of inflammatory cells into the renal parenchyma occurs early in the course of AKI. Macrophages are key effectors of the inflammatory cascade in various kidney injury models. Consistent with decreased pro-inflammatory cytokine production following rAAV9-IL-9 treatment, macrophage infiltration was measured by immunohistochemistry analysis. As shown in Figure 3A, in vehicle mice, CP injection produced a large increase in macrophages within the kidney cortex. In contrast, AAV9-IL9 mice had little or no impact of macrophage numbers.

To explore the role of IL-9 on pro-inflammatory cytokine secretion *in vitro*, THP-1 cells or RAW264.7 cells were harvested 8 h after induction with different doses of CP. The level of TNF-α was significantly increased following a dose of 100 μmol/L CP, which did not induce apoptosis in these cells (Supplementary Figures 1, 2). As noted above, we found that the level of IL-9 dramatically decreased 3.6-fold in CP nephrotoxicity in the supernatant of THP-1 (Figure 3B), consistent with an 8-fold sharp decrease in the mRNA level of IL-9 (Figure 3C). Similar results were observed in the level of IL-9 in RAW264.7 cells (Figures 3D,E).

To determine if secretion of inflammatory cytokines in macrophages was driven by IL-9, we determined the effect of IL-9 on CP-induced cytokine expression with 100 ng/ml rIL-9 on CP-induced THP-1 cells. Consistent with our THP-1 cell data, the expressions of TNF-α and IL-6 were clearly upregulated 3-fold and 1.3-fold (Figures 3F,G), respectively, in the CP-treated group, but these increases were significantly blunted with rIL-9 treatment in RAW264.7 cells (Figures 3H,I).

### Interleukin-9 Suppresses Injury of Tubular Epithelial Cells by Downregulation of Pro-Inflammatory Cytokines

**Figure 4:**
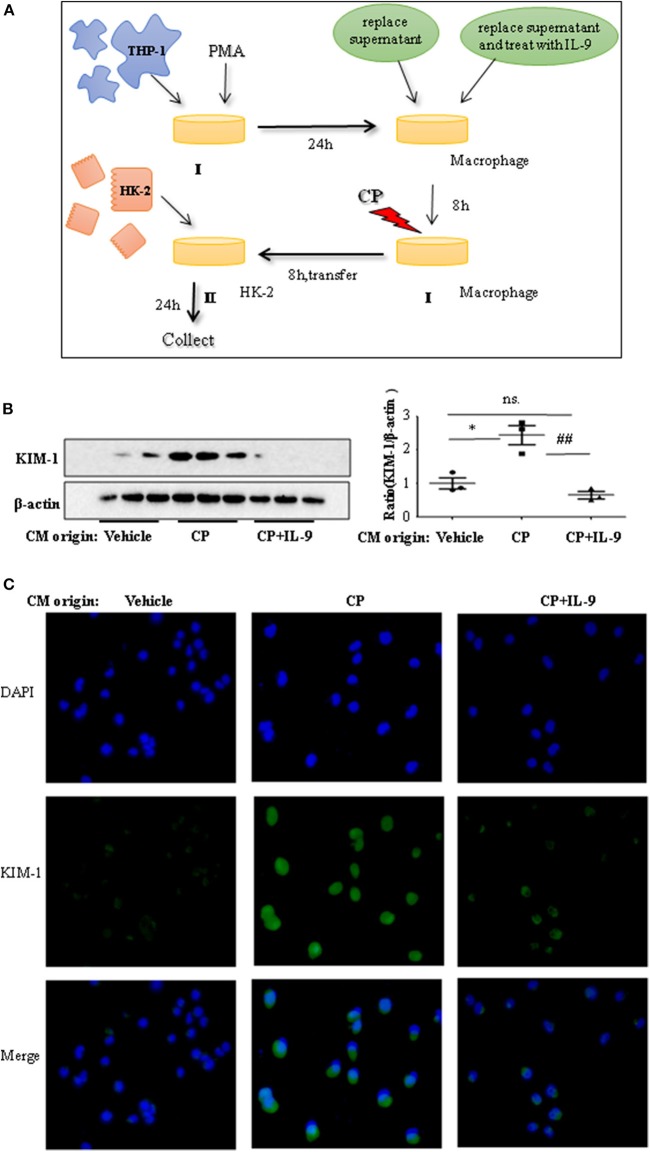
Interleukin-9 (IL-9) suppressed the kidney injury molecule-1 (KIM-1) expression of renal tubular epithelial cells with THP-1 conditioned medium (CM). Data were represented as mean ± SD of three independent experiments. **(A)** The coculture was set up as follows: THP-1 cells were seeded in the presence of phorbol 12-myristate 13-acetate (PMA) (100 nmol/L) to let them differentiate into macrophages. After 24 h, replace supernatant or replace supernatant and treat with IL-9 (100 ng/ml) for 8 h, then treat with cisplatin (CP) (100 μmol/L). Eight hours later, transferred the supernatant to HK-2 cells. Collected the supernatant and cells (CM) for further test. **(B)** Western blot was performed in HK-2 cell lines incubated with CM to determine KIM-1 expressions. Each lane represents one sample from an individual mouse. Dot plots show the corresponding quantification of band intensity and fold change. Western blot analysis of KIM-1 expression in HK-2 cell lines incubated with CM, and data are represented as mean ± SD of three independent experiments. *P < 0.05 vs. the vehicle group; ^##^*P* < 0.01 vs. the CP-treated group. **(C)** Representative images of KIM-1 immunohistochemical staining in HK-2 cells with different CM.

To confirm whether downregulation of pro-inflammatory cytokines was driven by the renoprotection of IL-9, a macrophage/epithelial CM culture model was established (Figure 4A). We performed a number of different coculture experiments with IL-9 or CP-tagged THP-1 cells and HK-2 cells: CM from THP-1 cells was added to HK-2 cells. In coculture conditions, the level of KIM-1 was increased in CP-induced CM compared to the normal HK2 cell line group. The suppressive effect of IL-9 and CP-induced THP-1 CM altered the level of KIM-1, with a 2.4-fold decrease (Figure 4B). A similar result was revealed following immunofluorescence staining of KIM-1 (Figure 4C).

### Histone Deacetylation Suppresses Interleukin-9 Following Cisplatin Administration *in vitro*

**Figure 5:**
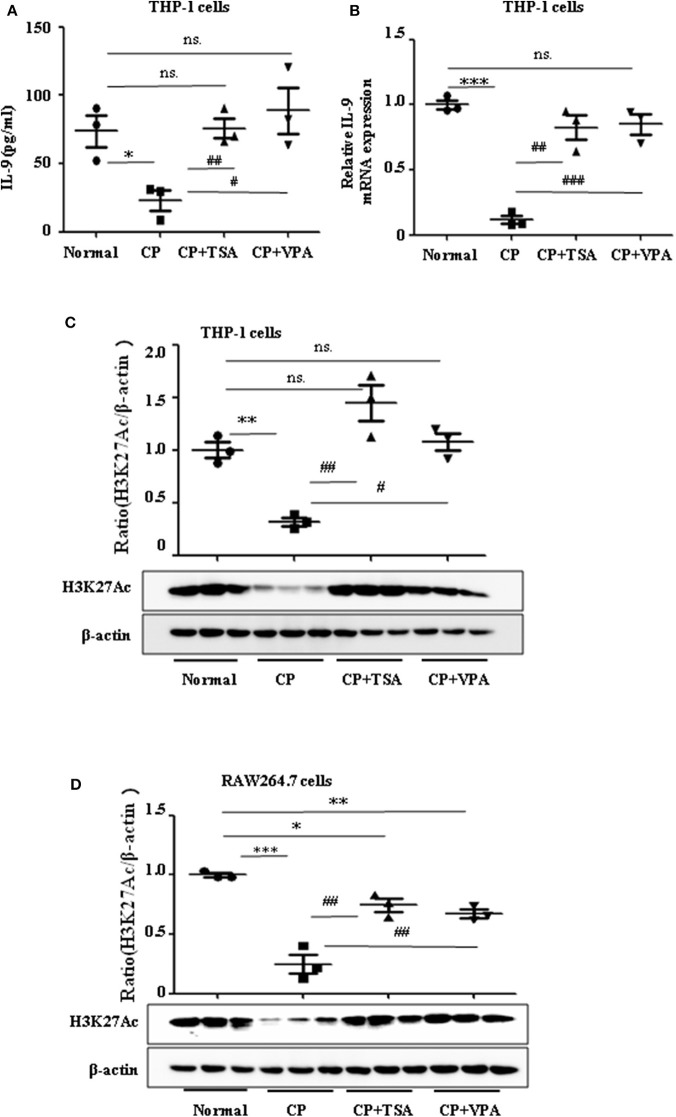
Trichostatin A (TSA) or valproate (VPA) upregulated interleukin-9 (IL-9) with cisplatin (CP) administration *in vitro*. Data are represented as mean ± SD of three independent experiments. **(A)** The level of IL-9 in CP-induced THP-1 cells with treatment of TSA (0.1 μmol/L) or VPA (1 mmol/L) respectively by ELISA. *P < 0.05 vs. the Normal group; ^##^*P* < 0.01, ^#^*P* < 0.05 vs. the CP-treated group. **(B)** The mRNA level of IL-9 in CP-induced THP-1 cells with treatment of TSA (0.1 μmol/L) or VPA (1 mmol/L), respectively. Dot plots show the corresponding quantification of mRNA level and fold change. ***P < 0.001 vs. the Normal group; ^###^*P* < 0.001, ^##^*P* < 0.01 vs. the CP-treated group. **(C)** Western blot was performed on CP-induced THP-1 cells with treatment of TSA (0.1 μmol/L) or VPA (1 mmol/L), respectively, to determine H3K27Ac expressions. Each lane represents one sample from an individual mouse. Dot plots show the corresponding quantification of band intensity and fold change. **P < 0.01 vs. the Normal group; ^##^*P* < 0.01, ^#^*P* < 0.05 vs. the CP-treated group. **(D)** Western blot was performed on CP-induced RAW264.7 cells with treatment of TSA (0.1 μmol/L) or VPA (1 mmol/L), respectively, to determine H3K27Ac expressions. Each lane represents one sample from an individual mouse. Dot plots show the corresponding quantification of band intensity and fold change. ***P < 0.001, **P < 0.01, *P < 0.05 vs. the Normal group; ^##^*P* < 0.01 vs. the CP-treated group.

Consistent with our previous data, we observed the ameliorative role of HDAC inhibitors on CP-induced AKI. However, more studies regarding the mechanisms of HDAC in AKI are needed, particularly as histone acetylation/deacetylation may contribute to renal damage. In this research, we found that HDAC inhibitors, TSA and VPA, significantly upregulated the expression of IL-9 in CP-induced THP-1 cells (Figures 5A,B), consistent with a recent report by Lloyd and Harker (18). Acetylation of histone H3 at lysine 27 (H3K27Ac) is a well-defined marker of enhancer activity. However, the functional impact of H3K27Ac in CP-induced AKI is poorly understood. Interestingly, we found that the protein level of histone H3K27Ac was downregulated in CP-induced THP-1 cells while TSA or VPA increased the protein level (Figure 5C). Consistent with THP-1 cell data, the protein level of H3K27Ac was also significantly decreased in CP-induced RAW264.7 cells but upregulated with HDAC inhibitor exposure (Figure 5D).

### Histone Deacetylation Suppresses Interleukin-9 With Cisplatin-Induced Renal Dysfunction

**Figure 6:**
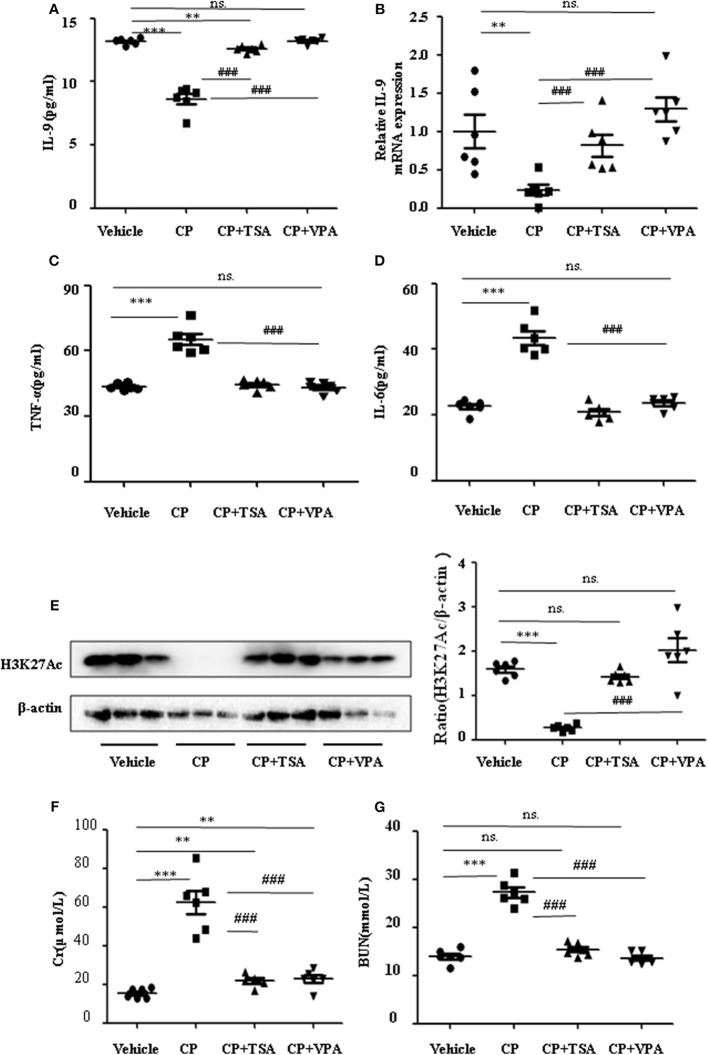
Trichostatin A (TSA) or valproate (VPA) upregulated interleukin-9 (IL-9) with cisplatin (CP)-induced acute kidney injury (AKI) mice *in vivo*. Three independent experimental series were performed with six animals per group in each series. Data are represented as mean ± SD (*n* = 6). **(A)** The level of IL-9 in CP-induced AKI mice with administration of TSA (1 mg/kg body weight) or VPA (1 mg/kg body weight), respectively, by ELISA. ***P < 0.001, **P < 0.01 vs. the vehicle group; ^###^*P* < 0.001 vs. the CP-treated group. **(B)** The mRNA level of IL-9 in CP-induced AKI mice with administration of TSA (1 mg/kg body weight) or VPA (1 mg/kg body weight), respectively. Dot plots show the corresponding quantification of mRNA level and fold change. **P < 0.01 vs. the vehicle group; ^###^*P* < 0.001 vs. the CP-treated group. **(C)** The levels of pro-inflammatory cytokine tumor necrosis factor-α (TNF-α) in serum of CP-induced AKI mice with administration of TSA (1 mg/kg body weight) or VPA (1 mg/kg body weight), respectively. ***P < 0.001 vs. the vehicle group; ^###^*P* < 0.001 vs. the CP group. **(D)** The levels of pro-inflammatory cytokine IL-6 in serum of CP-induced AKI mice with administration of TSA (1 mg/kg body weight) or VPA (1 mg/kg body weight), respectively. ***P < 0.001 vs. the vehicle group; ^###^*P* < 0.001 vs. the CP group. **(E)** The protein expression of H3K27Ac in CP-induced AKI mice with administration of TSA (1 mg/kg body weight) or VPA (1 mg/kg body weight), respectively. ***P < 0.001 vs. the vehicle group. **(F)** Changes in serum creatinine (Cr) level with CP-induced AKI mice with administration of TSA (1 mg/kg body weight) or VPA (1 mg/kg body weight), respectively. ***P < 0.001, **P < 0.01 vs. the vehicle group; ^###^*P* < 0.001 vs. the CP group. **(G)** Changes in serum blood urea nitrogen (BUN) level with CP-induced AKI mice with administration of TSA (1 mg/kg body weight) or VPA (1 mg/kg body weight), respectively. ***P < 0.001 vs. the vehicle group; ^###^*P* < 0.001 vs. the CP group.

**Figure 7:**
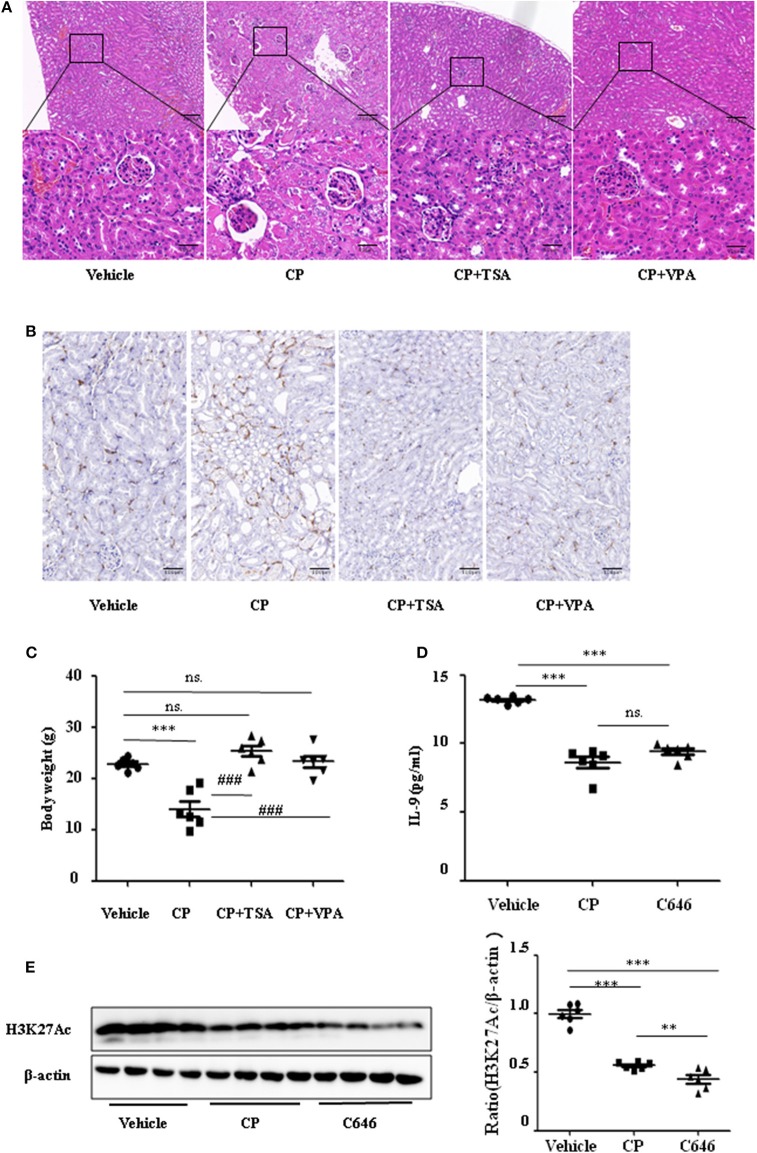
Histone deacetylase (HDAC) inhibitors suppressed interleukin-9 (IL-9) with cisplatin (CP) administration *in vivo*. Three independent experimental series were performed with six animals per group in each series. Data are represented as mean ± SD (*n* = 6). **(A)** Representative images of H&E staining of kidney section with CP-induced acute kidney injury (AKI) mice with administration of trichostatin A (TSA; 1 mg/kg body weight) or valproate (VPA; 1 mg/kg body weight), respectively. ***P < 0.001 vs. the vehicle group; ^###^ *P* < 0.001 vs. the CP group. Magnification: ×10 and ×40. **(B)** Representative images of Immunohistochemistry staining infiltrated macrophages. Magnification: ×40. **(C)** Body weight in each group during injection. ***P < 0.001 vs. the vehicle group; ^###^ *P* < 0.001 vs. the CP group. **(D)** The level of IL-9 in CP-induced AKI mice with administration of C646 by ELISA. ***P < 0.001 vs. the vehicle group. **(E)** Western blot was performed on CP-induced AKI mice with administration of C646 to determine H3K27Ac expressions. Each lane represents one sample from an individual mouse. Dot plots show the corresponding quantification of band intensity and fold change. ***P < 0.001, **P < 0.01 vs. the vehicle group.

To address the role of histone deacetylation in the pathogenesis of CP-induced acute renal failure, renal function was measured in animals treated with CP in the presence or absence of HDAC inhibitors, TSA, or VPA. TSA and VPA significantly upregulated the expression of serum IL-9 in CP-injected mice (Figures 6A,B). The expression of TNF-α and IL-6 was increased 72 h after CP administration, but these increases were significantly blunted in TSA- or VPA-treated mice (Figures 6C,D). In contrast, the protein level of histone H3K27Ac was suppressed in CP-induced mice but increased following treatment with TSA or VPA (Figure 6E). As expected, CP-induced mice developed severe kidney dysfunction, as reflected by elevated Cr and BUN concentrations. The more effective of these agents, TSA or VPA, reduced urea concentration by over 50% (Figures 6F,G). Similar results were found in H&E-stained kidney sections (Figure 7A) and body weight measurements (Figure 7C). The improvement in renal function was also reflected in less severe histologic damage. Immunohistochemistry analysis revealed that CP injection produced a large increase in macrophages. In contrast, mice treated with TSA or VPA had little or no increase in macrophages (Figure 7B).

Furthermore, we chose a specific histone acetylase inhibitor C646 to estimate the relationship between H3K27Ac and IL-9. Contrary to TSA and VPA, C646 significantly downregulated levels of IL-9 and H3K27Ac (Figures 7D,E), which indicates that downregulation of IL-9 was suppressed by histone deacetylation.

## Discussion

Inflammation is a complex biological response that is essential for eliminating microbial pathogens and repairing tissues after injury. AKI is associated with intra-renal and systemic inflammation. Thus, improved understanding of the cellular mechanisms underlying the inflammatory response has the potential to identify effective therapies to ameliorate AKI (6, 24, 25). It was demonstrated that macrophages are a major contributor to the inflammatory response in the AKI animal model. Emerging data from human biopsies also corroborate the presence of macrophages in AKI and their persistence in progressive chronic kidney disease (26–28). Resolution of inflammation is an attractive prospect for the treatment of AKI, but previously, little was known regarding the involvement of cytokines in this pathology. In this study, we demonstrate that IL-9 suppresses the secretion of pro-inflammatory cytokines in CP-induced AKI *in vivo*. Similarly, the secretion of pro-inflammatory cytokines was downregulated by rIL-9 in both THP-1 and RAW264.7 cell lines.

Accumulating evidence indicates that IL-9 plays an important role in diseases. Nevertheless, previous studies into the role of IL-9 in disease pathogenesis are conflicting. For example, Zhang et al. (29) observed that IL-9 promotes endothelial cell apoptosis, while Fontaine et al. (30) report that IL-9/IL-9 receptor signaling selectively protects cortical neurons against developmental apoptosis. Hu et al. (31) found that the local IL-9 level is significantly elevated in WT mice with ischemia–reperfusion (I/R) injury and consistent with the expressions of pro-inflammatory cytokines. Turner et al. (32) also reported that IL-9 promotes inflammation by directly enhancing the secretion of pro-inflammatory cytokines and chemokines by resident cells. However, Kortekaas et al. (33) showed that inhibition of IL-9 aggravates kidney damage in clinical I/R injury. Moreover, Eller et al. (34) provided the first direct *in vivo* evidence that the nephroprotective, anti-inflammatory effects of T regulatory cells (Tregs) critically depend on the mediating of IL-9. Rauber et al. (12) found that IL-9 restores homeostasis following inflammation, and that IL-9 treatment attenuates inflammation in a mouse model of arthritis. Cully (35) also reported that IL-9 resolves inflammation. As these many reports are conflicting, we focused on the contribution of IL-9 in CP-induced AKI. In this study, IL-9 was downregulated in CP-induced AKI. We found that the secretion of pro-inflammatory cytokines associated with macrophages was suppressed by overexpression of IL-9 *in vitro*. The CM from THP-1 cells that overexpressed IL-9 had a suppressive effect of injury of tubular epithelial cells. Renal dysfunction, tubule injury, and levels of serum urea and serum Cr were significantly attenuated in IL-9 overexpression AAV9-treated AKI mice. Our results indicate that IL-9 has a protective role in CP-induced AKI. Thus, we could improve the resolution of inflammation and reduce kidney damage *via* inhibiting the decrease of IL-9. This may provide a new route for the treatment of CP-induced kidney inflammation.

In allergic lung inflammation, Xiao et al. (19) found that the acetylation of histones (specifically, H3K27) mediates IL-9 expression. Lloyd and Harker (18) also reported that acetylation of histones mediates IL-9 in asthma, and inhibition of H3K27 acetylation decreases the expression of IL-9. Available evidence suggests that levels of HDAC enzymes closely correlate with cell proliferation and apoptosis. It was previously suggested that HDAC enzymes balance the acetylation activities of histone acetyltransferases in chromatin remodeling and play essential roles in regulating gene transcription. Previously, we found that administration of HDAC inhibitors, TSA or VPA, suppressed renal tubular epithelial cell apoptosis and may be responsible for promoting renal regeneration and functional recovery in AKI induced by CP (15). In this study, we observed that the levels of IL-9 are significantly decreased following CP injection, but these decreases are significantly blunted by the HDAC inhibitors, TSA, VPA, and C646. Furthermore, the expression of H3K27Ac was significantly upregulated with TSA, VPA, and C646 treatment. Inhibition of H3K27 acetylation also decreased the expression of IL-9. Thus, our results show that inhibition of H3K27 acetylation orchestrates IL-9-mediated renoprotection in CP-induced AKI.

**Figure 8:**
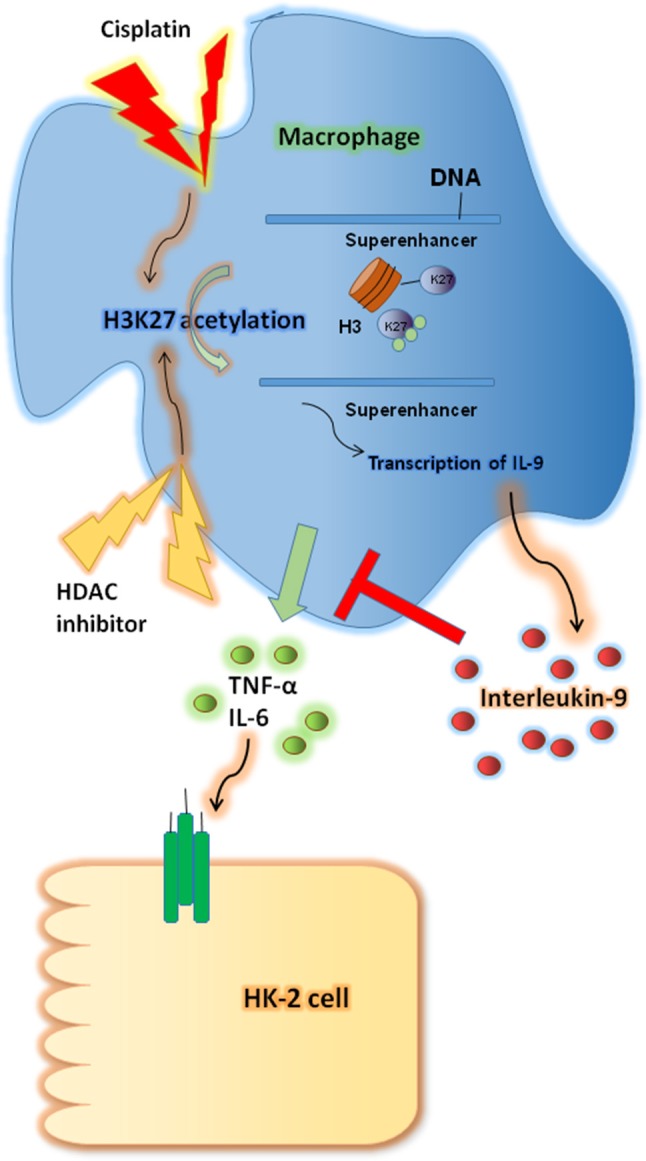
Renoprotective impact of interleukin-9 (IL-9) in cisplatin (CP)-induced acute kidney injury may be attributed to changes in renal function supported a regulatory, anti-inflammatory immune response. IL-9 suppressed pro-inflammatory cytokines such as IL-6 and tumor necrosis factor-α (TNF-α) associated with macrophages, and H3K27 acetylation promoted IL-9 secretion. Thus, inhibition of H3K27Ac orchestrated IL-9-mediated renoprotection in CP-induced acute kidney injury.

In this study we investigated the renoprotective effects of IL-9 in CP-induced AKI. IL-9 suppressed the pro-inflammatory cytokine levels associated with macrophages. Furthermore, H3K27 acetylation promoted IL-9 secretion. Depletion of H3K27 acetylation largely abolished the nephroprotective effects of IL-9 (Figure 8). Accordingly, our findings shed light on novel and anti-inflammatory properties of IL-9 that confer preservation of kidney function and structure in kidney inflammation and may counteract kidney disease procession.

## Data Availability Statement

All datasets generated for this study are included in the article/Supplementary Material.

## Ethics Statement

All animal procedures were approved by the Animal Experimentation Ethics Committee from Anhui Medical University, Anhui, China (LLSC20190277).

## Author Contributions

CHu performed the experiments and wrote the manuscript. TM conceived the idea of the study, designed the experiments, and wrote and edited the manuscript. WJ and XY designed and performed the experiments. HZ, CHe, CG, QT, CX, and BH performed the experiments.

### Conflict of Interest

The authors declare that the research was conducted in the absence of any commercial or financial relationships that could be construed as a potential conflict of interest.
