# Supplementary material for: Inhibition of Histone H3K27 Acetylation Orchestrates Interleukin-9-Mediated and Plays an Anti-Inflammatory Role in Cisplatin-Induced Acute Kidney Injury
Source: Front Immunol. 2020 Mar 3;11:231. doi: 10.3389/fimmu.2020.00231 (PMC7062682; doi:10.3389/fimmu.2020.00231)
Supplement: Supplementary file 1 [file Data_Sheet_1.pdf]

Supplementary Material

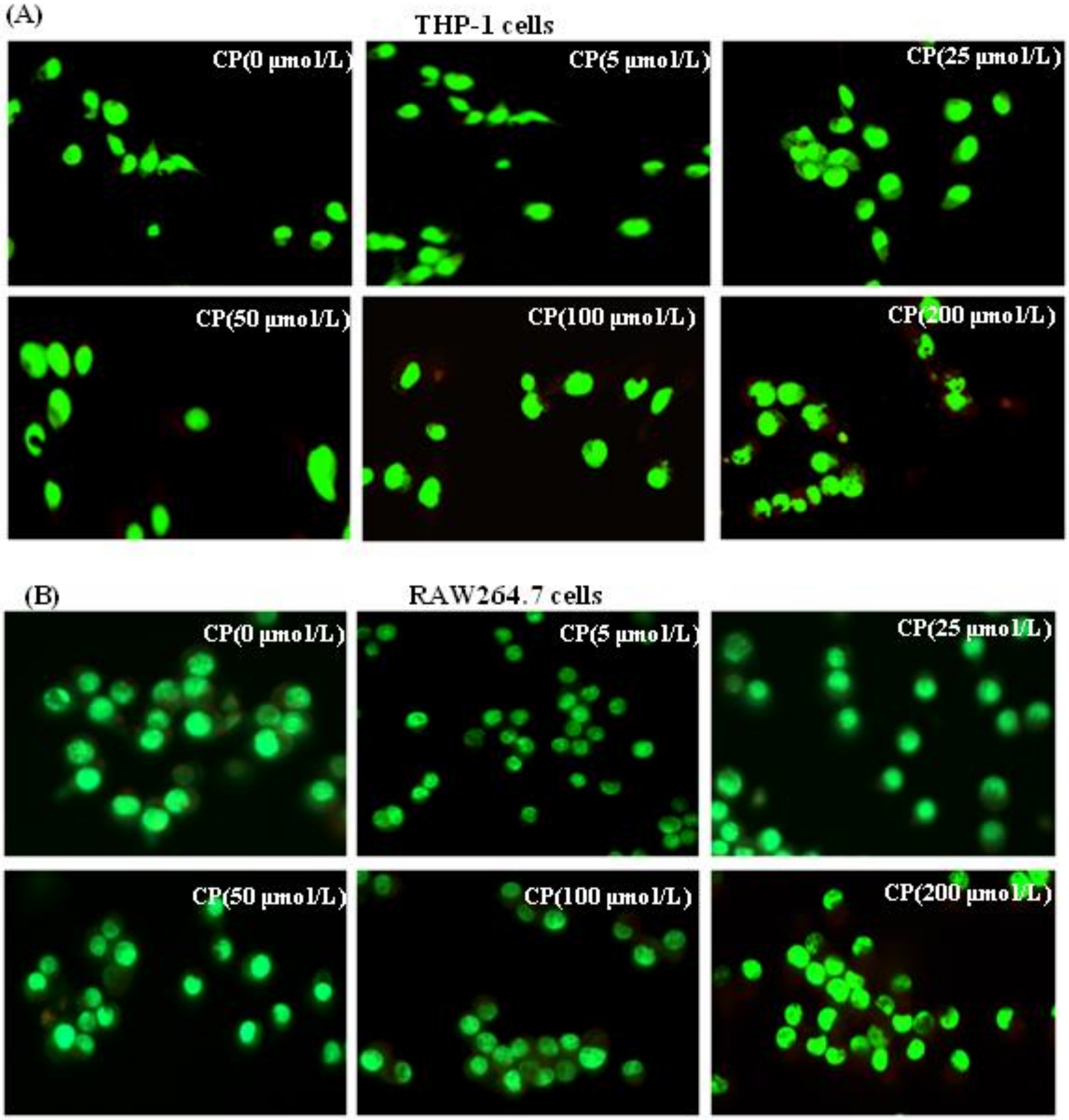

Supplementary Figure 1 Different dose of CP (0-~200μmol/L) induced the cell injury not apoptosis with fluorescent staining *in vitro.*

(A) Orange-ethidium bromide (AO/EB) fluorescent staining of THP-1 cell lines with different dose of CP (0-~200μmol/L).

(B) Orange-ethidium bromide (AO/EB) fluorescent staining of RAW264.7 cell lines with different dose of CP (0-~200μmol/L).

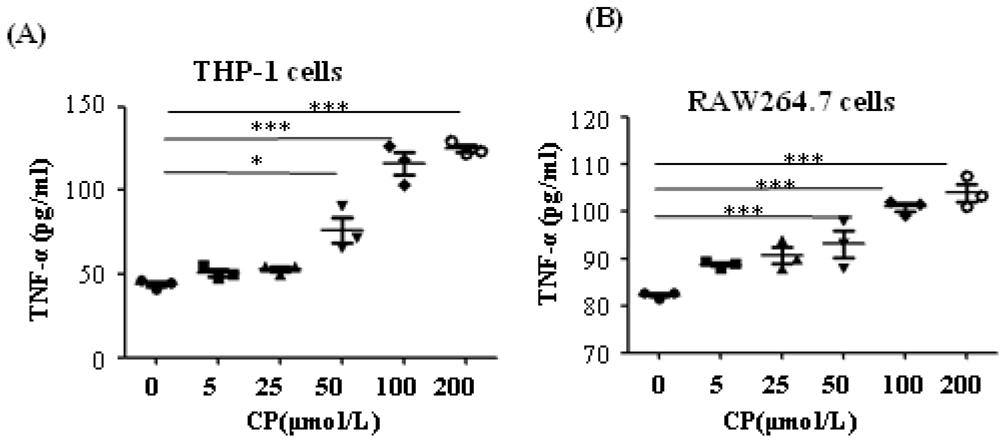

Supplementary Figure 2 The secretion of pro-inflammatory cytokines with different dose of CP administration *in vitro.*

(A) The level of TNF-α in THP-1cells with different dose of CP administration. *P<0.05, ***P<0.001 versus CP (0 μmol/L) group. All experiments were performed in triplicate.

(B) The level of TNF-α in RAW264.7 cells with different dose of CP administration. ***P<0.001 versus CP (0 μmol/L) group. All experiments were performed in triplicate.
